# The severe impact of the COVID-19 pandemic on bullying victimization, mental health indicators and quality of life

**DOI:** 10.1038/s41598-022-27274-9

**Published:** 2022-12-31

**Authors:** June T. Forsberg, Steinar Thorvaldsen

**Affiliations:** 1 Regional Center for Child and Adolescent Mental Health (RKBU North), UiT, The Arctic University of Norway, Tromsö, Norway; 2 Department of Education, UiT, The Arctic University of Norway, Tromsö, Norway

**Keywords:** Psychology, Human behaviour

## Abstract

Children and adolescents have been severely affected by the COVID-19 pandemic. The aim of this study was to explore the prevalence of traditional and digital bullying and mental health problems a year into the pandemic. Further, how anxiety level, loneliness, and self-perceived school functioning have influenced the quality of life. A comprehensive questionnaire was administered (N = 1239) in the city of Tromsø and compared to a similar data collection (N = 972) conducted in the same schools in 2017. The main findings were increased prevalence in bullying, more mental health problems and significantly reduced quality of life compared to before the pandemic. Implications and the importance of implementing anti-bullying and psychosocial measures after the pandemic are discussed.

## Introduction

On March 12, 2020, the day after the World Health Organization (WHO) declared the spread of SARS-CoV-2 as a pandemic, Norway and several other countries around the world locked down large parts of society in attempt to limit the spread of the virus. The lockdown has been referred to as the most drastic action carried out in Norway since World War II^1^. The measures that were taken included isolation and quarantine regulations, comprehensive social distancing, and total lockdown of schools, public offices and services, and leisure activities for a period of 2 months. The lockdown led to concerns about the impact on children and adolescents’ well-being, quality of life (QoL), and possible mental health consequences, and researchers all over the world engaged in comprehensive work to document possible negative impacts^2–7^. The concerns were found to be legitimate. In Europe, children and adolescents reported reduced QoL, higher anxiety levels, more depression symptoms, loneliness, and behavioral problems in several studies, already after the first 3 months of the pandemic^4–6,8^.

Ravens-Sieberer et al.^6^ also found that two-thirds of their participants reported to be highly burdened by the pandemic. A specific burden was the reduced ability to socialize with friends and peers. For children and adolescents are socialization and positive relations with peers of high importance for general well-being^9^, QoL^10^, development of social skills^11^, and the ability to form positive relations when they grow up^12^. In addition, these relations are protective factors against the negative impact of stress^13^ and mental health problems^14,15^. On the other hand, problems with peers predict an increased risk of developing mental health issues and behavioral problems both during childhood, adolescence, and later in life^12^.

The reduced ability to socialize increased the prevalence of loneliness within all age ranges, and the pandemic has also been referred to as “the loneliness pandemic” in the literature^16^. Loneliness is associated with reduced QoL^17^ and is found to predict mental health problems and psychiatric impact, such as depression symptoms, social anxiety, paranoia, and suicide attempts. During the lockdown, the adolescents who felt lonely also reported that they experienced more mental health problems^8,18,19^. Without support from family, friends, and clinical measures (if necessary), have mental health symptoms the tendency to increase with age and into adulthood. The importance of follow-up and support for vulnerable young people after the pandemic is therefore highlighted in the literature^8^.

When the second virus wave hit Norway during the fall of 2020, was the city of Tromsø obliged to reinforce lockdown in some parts of society^1^. In attempt to spare children and adolescents for the negative psychosocial consequences of lockdown, the schools were kept open. However, strict infection control measures were employed, which had severe influence on the daily routines at the schools. The measures were implemented as a traffic light model, with three levels of measures (red, yellow, and green). Avoidance of all physical contact was practiced at all three levels. In levels yellow and red, the pupils were divided into cohorts (different cohort sizes depending on the level of measure) and practiced both social and physical movement restrictions. When a pupil tested positive for COVID-19, the rest of the cohort was placed in quarantine, and homeschooling was initiated for 10 days. The traffic light model was maintained throughout the second and third virus waves, until the end of May 2021^20^, and the level of measure implemented was under continuous consideration depending on the extent of virus spread at each school^21^. In Tromsø, level yellow was maintained for most of the time during this period. Although the traffic light model was important to limit the spread of the virus in the schools, it has also been a disturbing and negative factor for the academic, psychosocial, and institutional environment at school. The quality of the overall school climate is an important predictor for children’s and adolescents’ ability to function at school^22^.

### Bullying during the pandemic

Bullying victimization refers to repeated acts where a person is exposed to intentional negative actions, such as verbal, physical, and/or relational aggression, or harassment, by a peer or a group of peers in real life or online^23,24^. A well-established time frame in the literature for conceptualizing these repeated negative acts as bullying, is if a person report that it happens “two or three times per month or more often”^24–26^. A recent national report shows that 6% of adolescents in Norway report that they are being bullied at school, and 3% report digital bullying^27^. Being bullied by peers has a major negative impact on psychosocial well-being and school functioning, and it is considered one of the most stressful life events that young people can be exposed to^12,28–30^. Bullying is often divided into four categories: (I) direct physical bullying (e.g., physical attacks), (II) direct verbal bullying (threats, insults, or name calling), (III) indirect verbal/social bullying (social exclusion or spreading of rumors), and (IV) cyberbullying/digital bullying or harassment (threatening messages, posting of pictures from vulnerable situations, exclusion from social media). The first three categories can be considered traditional bullying, whereas digital bullying is a phenomenon that emerged as a result of the technological era and is relatively new in the research field. Therefore, the negative impact of digital bullying compared to traditional bullying has been a focus in several studies in recent decades^23,25,26^. The literature reports that being harassed on digital platforms or being a victim of cyberbullying is associated with the same mental health problems and psychosocial consequences as traditional bullying^31^. During the pandemic, technological tools and digital social platforms were highlighted as measures to reduce loneliness^32^, but, unfortunately, there has also been documented an increased prevalence of digital bullying^33,34^.

In the work of documenting the prevalence of bullying during the pandemic, the literature is inconclusive. Vaillancourt et al.^35^ explored both bullying at school and digital bullying before and 6–8 months into the pandemic. They found that the participants reported more bullying of all forms before the pandemic, except for digital bullying, where the differences were less pronounced. The conclusion of the study was that the pandemic in general seems to have had a positive effect on bullying rates. Armitage^36^, on the other hand, raises a major concern that bullying may have substantially increased during the pandemic, due to less focus on anti-bullying measures and more opportunities for digital bullying. Further, he states that there is an urgent need to determine the impact of the pandemic on the prevalence of all forms of bullying and for implementation of measures and interventions to reduce the negative impact on children and adolescents’ health.

### Objectives of the present study

The aims of this study were to investigate the impact of COVID-19, a year into the pandemic, on bullying; emotional, behavioral, and peer problems; and QoL among children living in Northern Norway. To the best of our knowledge, no studies have explored these factors at this stage in the pandemic. The research tasks were as follows:

1. Explore the prevalence of traditional and digital bullying during the COVID-19 pandemic compared to before the pandemic.
2. Explore whether emotional, behavioral, and peer problems increased during the COVID-19 pandemic compared to before the pandemic.
3. Explore how anxiety level, loneliness, and self-perceived school functioning during the COVID-19 pandemic impacted the subjective feeling of life quality.

All research tasks also explored gender differences and differences between age groups. Based on Barlett et al.^33^, Shin & Choi^34^ and Vaillancourt et al.^35^ we hypothesized reduced traditional bullying and increased digital bullying during the pandemic compared to before. Further, we expected to find increased scores in emotional problems, behavioral problems, and peer problems^4–6,8^. We also expected to find reduced QoL due to anxiety^4^, loneliness^17^, and self-perceived school functioning^22^ during the pandemic.

### Method

The analysis presented in this article is part of the project *Well-being in Tromsø* (WiT) (org. title: Trivsel i Tromsø) of UiT the Arctic University of Norway. WiT collected data on bullying, harassment, mental health, and QoL once a year during the period of 2012–2018 and WiT-2 in 2021, in the same school and the same grades. Although the WiT project is longitudinal, no longitudinal analyses of the same children was carried out in the present study.

The data that describe the **pandemic group** were collected in April 2021, a year into the pandemic (N = 1239: 573 females and 666 males). The data that describe the **pre-pandemic group** were collected in the spring of 2017 (N = 972: 452 females and 520 males). Data from 2017 were chosen instead of data from 2018 due to better quality and higher response rate (65% and 48%, respectively).

### Participants, design, and procedure

A total of 2,211 school children (1,025 females and 1,186 males) between grades 4 and 10 (ages 9–15) were included in the analysis for the present study. The participants were split into three different age groups: grades 4 + 5 (middle school grades/age 9–10 years old) (N = 741: 355 females and 386 males); grades 6 + 7 (upper school grades/age 11–12 years old) (N = 757: 348 females and 409 males); and grades 8–10 (junior high grades/age 13–15 years old) (N = 713: 322 females and 391 males). The response rates were 85% for the pandemic group and 65% for the pre-pandemic group. There were no significant differences between genders (*p* = 0.25) or age groups (*p* = 0.87) in the pandemic and pre- pandemic groups.

A cross-sectional comparative design with two groups (pandemic group and pre-pandemic group) was employed for all analysis in this study. The procedure was similar for both groups, but with some points of distinction. The study was presented and proposed to the school leaders in the WiT project, which agreed that the study could be conducted in their schools (N = 6). Thereafter were the teaching staff at each school invited to meetings of 30 min (physical meetings in 2017 and digital meetings in 2021) were the purpose of the study and procedure were presented, and the teachers could ask questions they might have. Further, study information was forwarded to the parents via a digital information channel (Transponder). In the pre-pandemic group, was personal information collected; therefore, required approval by the Regional Ethical Committee for Medical Research, REK-Nord, and written consent by the parents. In the pandemic group were no personal data collected, and participation was anonymous; therefore, no ethical was required.

The data collection was carried out digitally in the classrooms using Questback (pre-pandemic group) and Nettskjema (pandemic group). Both Questback and Nettskjema is commercial tools developed for use in a wide range of investigations and has good reputation for data security. However, due to changes in UiT license agreements, Questback was replaced with Nettskjema between the data collections. Before the pupils filled out the questionnaires they were informed of the purpose of the study and some basic research ethical principles, i.e., that participation is voluntarily, and that all data are kept safe and only accessible for the researchers. In the pre-pandemic group this information was provided by the teachers. In the pandemic group did the researchers prepare a short video where the purpose and ethics were presented. Since participation was anonymous in this group, this was also emphasized in the video. The data collections were carried out over a period of 2 months during the spring of 2017 and 2021.

### Measures

WiT uses several measures to gain information on school children’s self-perceived understanding of the impact of COVID-19 on bullying (both traditional and digital); emotional, behavioral, and peer problems; and the subjective feeling of life quality. A comprehensive questionnaire was carried out that included several sets of measures. Bullying, the Strength and Difficulties Questionnaire (SDQ), and KINDL® (description below) were employed as measures for all participants included in this study. The COVID categories were employed in the pandemic group only. All items and categories in the questionnaire are presented in Online Appendix1.

#### Bullying

Bullying was measured with 15 items for traditional bullying^25,26^ and explored verbal (five items), social (six items), and physical bullying (four items). Further, digital bullying was measured with eight items from Menesini et al. ^23^. The items employed a five-point Likert scale (1 = “never”, 2 = “only once or twice”, 3 = “two or three times a month”, 4 = “about once per week”, and 5 = “several times a week). The time frame was set for the past 3 months. The cut-off point for being bullied was set to “two or three times a month” or more often^24–26^. Cronbach’s alpha for the different scales is consistent, with high reliability (verbal α = 0.84, social α = 0.85, physical α = 0.86, and digital α = 0.87). Prevalence of bullying was measured with two items (I am being bullied during the school day *and* I am being bullied outside of school), and max scores were calculated for traditional bullying, digital bullying, and both.

#### Strength and difficulties questionnaire (SDQ)

The SDQ^37,38^ is comprised of five scales of five items each that assess emotional problems (α = 0.66), conduct problems (α = 0.60), hyperactivity (α = 0.67), peer problems (α = 0.41), and prosocial skills (α = 0.66). The reliability is below the cut-off of 0.7 for obtaining acceptable reliability for all five scales, but the SDQ has been a widely used tool since it was developed^39,40^. The items are scored on a three-point Likert scale (“not true”, “somewhat true”, “certainly true”). The time frame was set for the past 3 months. “Somewhat true” is always scored as 1, but the scoring of “not true” and “certainly true” varies depending on whether the item is positively or negatively worded. For each of the five scales, the score can range from 0 to 10, if all items are completed. The total difficulties scale (α = 0.80) is generated by summing scores from all the scales, except the prosocial scale. The result score for the total difficulties scale can range from 0 to 40 and is counted as missing if one of the component scales is missing. The prosocial scale was not included in any analysis for the present study.

#### KINDL®

KINDL®^41,42^ measures experienced QoL and consist of a 24-item scale (α = 0.84) that measures six different dimensions (subscales) of QoL: experienced physical health (α = 0.63), emotional well-being (α = 0.68), self-esteem (α = 0.75), relationship to family (α = 0.76), relationship to friends (α = 0.74), and relationship to school (α = 0.64). Every question asks about the previous week’s experiences and is scored on a five-point Likert scale (1 = “never”, 2 = “rarely”, 3 = “sometimes”, 4 = “often”, and 5 = “always”). Ten of the QoL items have reverse-order scaling, meaning that a higher item score implies poorer QoL, and these item scores were reversed. Mean item scores are calculated for all the subscales. Correlations with comparable QoL scales have shown acceptable reliability as well as satisfactory discriminant validity^43^.

#### COVID categories

The impact of the COVID-19 pandemic was measured with three categories that were developed for this study: **anxiety** (three items: Have you been worrying about the virus? | Do you think about the virus even when you don’t mean to? | Have you been afraid to be infected when you are at school? | α = 0.76), **loneliness** (two items: Have you been with your friends as much as you have wanted to? | Have you been lonely? | α = 0.73), and **school functioning** (three items: Have you been able to concentrate on your schoolwork? | Did you manage to concentrate when there was homeschooling? | Have you been able to do your best with your schoolwork? | α = 0.73). The items were scored on a five-point Likert scale (1 = “never”, 2 = “rarely”, 3 = “sometimes”, 4 = “often”, and 5 = “always”). Four items have reverse-order scaling, meaning that a higher item score implies poorer circumstances.

### Statistical analysis

All statistical analyses were conducted using SPSS 28.0 (SPSS, Inc., Chicago, IL. USA). Reliability for all measurement scales was explored with Cronbach`s alpha. For the first research task, the prevalence of traditional and digital bullying was explored with frequency statistics. Differences in prevalence between the pandemic group and the pre-pandemic group and differences between genders were tested with a chi-square test. Differences in prevalence between age groups was explored with analysis of variance (ANOVA). Significant effects were followed up with contrast analysis, and the least significant difference (LSD) adjustment was chosen for the multiple comparisons. Effect sizes were estimated by Cohen’s d-value, with Cohen’s conventions: 0.2 = small effect, 0.5 = medium effect, and 0.8 = large effect^44^. Cohen’s d expresses the standardized difference between two means.

For the second research task, emotional problems, behavioral problems, and peer problems between the pandemic group and the pre-pandemic group were explored with descriptive statistics and multivariate analysis of variance (MANOVA). Gender and age groups were included as covariates in the analysis. Significant effects were followed up with LSD adjustments. Wilks’s lambda was conducted to determine if there were differences between the groups in these combinations of dependent variables.

For the third research task, the COVID categories (anxiety, loneliness, and self-perceived school functioning) were explored as dependent variables in three separate linear multiple regression analyses. The KINDL subscales and total sum scale were included as independent variables. However, self-esteem (β = 0) was excluded as a variable in all three regression models. Correlations between all the dependent and independent variables were also explored. Pearson’s r-coefficient was used to express the strength of correlation between two variables. Typically, values of ± 0.1 represent a small effect, ± 0.3 a medium effect, and ± 0.5 a large effect^44^. A significant *p*-value of 0.05 was set for all analyses in this study.

### Ethics approval and consent to participate

This study was conducted according to the principles of the Declaration of Helsinki. Written information about the project was provided to the students and parents, and the parents gave consent for their children to participate. Both the students and parents were able to withdraw consent at any time and require that collected data would be deleted. The authors were the only ones with access to the data during the data collections. Data collection 2021: No personal data were collected; therefore, no ethical approval was required. Data collection 2017: Personal information was collected; The project was approved by Regional Ethical Committee for Medical Research, REK-Nord. However, no personal data from the data collection in 2017 was included in this study.

A contract was made between UiT the Arctic University of Norway and the school leaders at each contributing school. The contract described area of responsibility and assignments for the school leaders, teachers, and researchers during the project period.

## Results

### Prevalence of traditional and digital bullying

Before the pandemic, 7.0% (68/972) of the participants reported that they experienced traditional bullying and 2.1% (20/972) reported digital bullying two to three times per month or more. 8.1% (79/972) reported either traditional bullying, digital bullying, or both. During the pandemic, the prevalence was found to be significantly increased to 10.1% (125/1239) for traditional bullying (X^2^ = 9.47, df = 4, *p* < 0.05) and 6.0% (74/1239) for digital bullying (X^2^ = 16.18, df = 4, *p* < 0.01). 11.9% (148/1239) reported that they experienced either traditional bullying, digital bullying, or both. This is significantly higher than before the pandemic (X^2^ = 14.26, df = 4, *p* < 0.01).

Before the pandemic, there was a significant gender difference (X^2^ = 11.69, df = 4, *p* < 0.05) in traditional bullying.4.5% (20/452) of the females and 9.3% (48/520) of the males reported traditional bullying. In digital bullying there was 1.5% (7/452) of the females and 2.8% (13/520) of the males that reported being bullied, thus there was no gender difference (X^2^ = 3.64, df = 4, *p* = 0.46). During the pandemic, 8.9% (51/573) of the females and 11.2% (74/666) of the males reported traditional bullying. Moreover, 3.9% (22/573) of the females and 6.4% (52/666) of the males reported digital bullying. There was no significant gender difference in either traditional or digital bullying during the pandemic (X^2^ = 2.54, df = 4, *p* = 0.64; X^2^ = 8.52, df = 4, *p* = 0.07, respectively).

**Figure 1:**
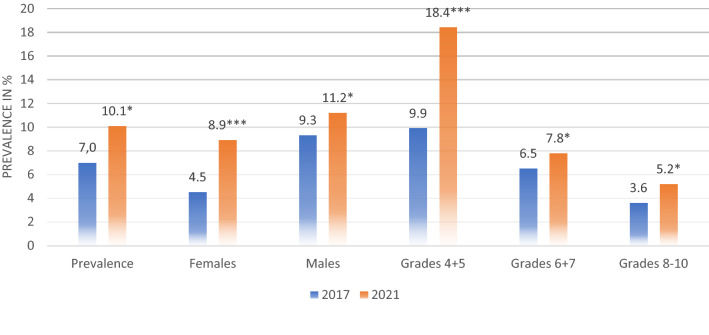
Prevalence of traditional bullying. *p < .05, **p < .01, ***p < .001.

**Figure 2:**
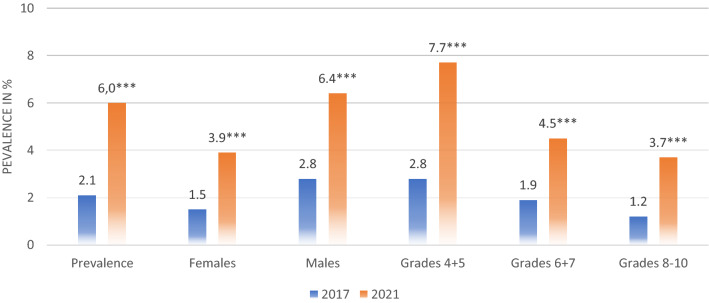
Prevalence of digital bullying. *p < .05, **p < .01, ***p < .001.

Before the pandemic, 9.9% (35/354) of the children in grades 4 + 5 reported traditional bullying. Further, 6.5% (24/370) in grades 6 + 7 and 3.6% (9/248) in grades 8–10 reported the same. There was a significant difference between the age groups (F [4,967] = 6.24, *p* < 0.001). Moreover, 2.8% (10/354) of the children in grades 4 + 5, 1.9% (7/370) in grades 6 + 7, and 1.2% (3/248) of the children in grades 8–10 reported digital bullying. There was a significant difference between age groups also in digital bullying (F [4,967] = 2.62, *p* < 0.05). During the pandemic, 18.4% (71/387) of the children in grades 4 + 5, 7.8% (30/387) in grades 6 + 7, and 5.2% (24/465) in grades 8–10 reported traditional bullying. There was an effect of age group (F [4,1234] = 14.36, *p* < 0.001), and grades 4 + 5 reported significantly more bullying compared to grades 8–10 (*p* < 0.001) and grades 6 + 7 (*p* < 0.001). Moreover, 7.7% (30/387) of the children in grades 4 + 5, 4.5% (17/387) in grades 6 + 7, and 3.7% (17/465) in grades 8–10 reported digital bullying. There was also an effect of age group on digital bullying (F [4, 1234] = 7.24, *p* < 0.001), and grades 4 + 5 reported significantly more bullying compared to the other age groups (*p* < 0.001). An overview of the prevalence of traditional and digital bullying is presented in Figs. 1 and 2.

### Emotional, behavioral, and peer problems

An effect of group was found in three of the four difficulties scales and for total difficulties (emotional problems: F (1,2209) = 91.58, *p* < 0.001; conduct problems: F (1,2209) = 8.69, *p* < 0.01; peer problems: F (1,2209) = 45.19, *p* < 0.001; total difficulties: F (1,2209) = 11.73, *p* < 0.001; Λ: F = (5,2203) = 1510.96, *p* < 0.001. The mean was higher in all these scales in the pandemic group compared to the pre-pandemic group. Gender was found to be a significant covariate in emotional problems: F = 30.87, *p* < 0.001; hyperactivity: F = 12.20, *p* < 0.001; and total difficulties: F = 44.02, *p* < 0.001 (Λ: F = 58.81, *p* < 0.001). Females reported significantly higher means in these three scales compared to males in both groups.

Age group was found to be a significant covariate in emotional problems: F = 59., *p* < 0.001; peer problems: F = 4.11, *p* < 0.05; and total difficulties: F = 22.77, *p* < 0.001 (Λ: F = 15.80, *p* < 0.001). Emotional problems were found to increase significantly with each age group (*p* < 0.001), and the problems were significantly higher in the pandemic group compared to the pre-pandemic group (*p* < 0.001). Peer problems were reported to be significantly higher in grades 4 + 5 in the pre-pandemic group compared to the other age groups (*p* < 0.05). In the pandemic group, peer problems were found to be significantly higher in grades 8–10 (*p* < 0.05). In total difficulties, grades 4 + 5 and grades 8–10 reported significantly higher scores than grades 6 + 7 in the pre-pandemic group. In the pandemic group, the score increased significantly with age (*p* < 0.001). An overview of all scale scores (mean and standard deviation (SD) and Cohens *d*) is presented in Table 1.

**Table 1:** Overview of all SDQ scale scores^§^.

Difficulties scale	Pre-pandemic group (N = 972)	Pandemic group (N = 1239)	Effect size				
	Mean (SD)	Mean (SD)	Cohens *d*				
Emotional problems***	0.44 (.42)	0.66 (.55)***	.42				
Conduct problems***	0.27 (.29)	0.36 (.32)***	.28				
Hyperactivity***	0.66 (.42)	0.79 (.43)***	.29				
Peer problems***	0.30 (.32)	0.45 (.39)***	.41				
Total difficulties***	8.44 (5.35)	11.29 (5.97)***	.50				

Gender	Pre-pandemic group (N = 972)		Pandemic group (N = 1239)		Effect size		
	Females (N = 452)	Males (N = 520)	Females (N = 573)	Males (N = 666)	Females/males		
	Mean (SD)	Mean (SD)	Mean (SD)	Mean (SD)	Cohens *d*		
Emotional problems***	0.52 (.44)***	0.38 (.39)	0.83 (.58)***	0.52 (.49)	.57/.30		
Conduct problems	0.21 (.24)	0.32 (.32)	0.34 (.31)	0.37 (.32)	.47/.15		
Hyperactivity***	0.64 (.43)***	0.68 (.43)	0.78 (.44)***	0.80 (.42)	.31/.26		
Peer problems	0.27 (.21)	0.33 (.33)	0.45 (.38)	0.45 (.40)	.51/.32		
Total difficulties***	8.22 (5.29)***	8.64 (5.39)	11.97 (6.19)***	10.70 (5.71)	.65/.37		

Age group	Pre-pandemic group (N = 972)			Pandemic group (N = 1239)			Effect size
	Grades 4 + 5 (N = 354)	Grades 6 + 7 (N = 370)	Grades 8–10 (N = 248)	Grades 4 + 5 (N = 387)	Grades 6 + 7 (N = 387)	Grades 8–10 (N = 465)	Grades 4 + 5/6 + 7/8–10
	Mean (SD)	Mean (SD)	Mean (SD)	Mean (SD)	Mean (SD)	Mean (SD)	Cohens *d*
Emotional problems***	0.42 (.38)	0.42 (.40)	0.53 (.49)***	0.52 (.49)	0.65 (.54)***	0.78 (.59)***	.24/.48/.45
Conduct problems	0.29 (.28)	0.27 (.231	0.25 (.28)	0.32 (.29)	0.38 (.32)	0.38 (.33)	.10/.36/.38
Hyperactivity	0.67 (.43)	0.61 (.42)	0.73 (.43)	0.74 (.43)	0.78 (.41)	0.83 (.43)	.16/.41/.23
Peer problems*	0.33 (.29)*	0.28 (.34)	0.30 (.32)	0.38 (.37)	0.48 (.40)	0.48 (.39)*	.15/.54/.50
Total difficulties***	8.57 5.06)***	7.91 (5.35)	9.05 5.68)***	9.85 (5.74)	11.49 (5.97)***	12.32 (5.94)***	.24/.63/.56

^§^Scale 0–2 for the subscales (emotional, conduct, hyperactivity, peer) and scale 0–40 for total difficulties. *p < .05, **p < .01, ***p < .001.

### The impact of COVID-19 variables on subjective life quality

#### Anxiety

Anxiety was found to significantly correlate in a negative direction with all QoL subscales. All correlations were represented by Pearson’s r below—0.3. The results from the multiple regression model indicated that the independent variables combined explained 8.6% of the variance (R^2^ = 0.086, F [6,1232] = 19.39, *p* < 0.001). All QoL subscales were found to be significant predictors.

#### Loneliness

Loneliness was found to significantly correlate in a positive direction with all QoL subscales. All correlations were represented by Pearson’s r above 0.3. Emotional well-being (r = 0.59, *p* < 0.001), relationship to friends (r = 0.51, *p* < 0.001), and total QoL (r = 0.58, *p* < 0.001) were found to be highly correlated with loneliness. The results from the regression model indicated that the independent variables combined explained 39.4% of the variance (R^2^ = 0.394, F [6,1232] = 133.47, *p* < 0.001). Emotional well-being (β = 0.37, *p* < 0.001) and relationship to friends (β = 0.23, *p* < 0.001) were found to be significant predictors alone.

#### School functioning

School functioning was found to significantly correlate in a negative direction with all QoL subscales. The results from the regression model indicated that the independent variables combined explained 33.9% of the variance (R^2^ = 0.339, F [6,1232] = 105.23, *p* < 0.001). Relationship to family (β = − 0.07, *p* < 0.05), relationship to friends (β = 0.13, *p* < 0.01), relationship to school (β = − 0.22, *p* < 0.001), and total QoL (β = − 0.48, *p* < 0.001) were found to be significant predictors alone. The results from the correlations and regression analysis are presented in Table 2. The correlations between the COVID-19 variables are presented in Table 3.

**Table 2:** Results from correlations and multiple regression analysis.

Variables	*r*	*p*	β	t	*p*
**COVID anxiety**					
Experienced physical health	− .19***	< .001	− .16***	− 3.20	< .001
Emotional well-being	− .25***	< .001	− .27***	− 4.87	< .001
Self-esteem	− .10***	< .001	-	-	-
Relationship to family	− .13***	< .001	− .11**	− 2.68	< .01
Relationship to friends	− .17***	< .001	− .15**	− 3.17	< .01
Relationship to school	− .24***	< .001	− .27***	− 5.35	< .001
Total QoL	− .24***	< .001	− .49***	− 3.68	< .001
**COVID loneliness**					
Experienced physical health	.38***	< .001	− .02	− .58	.56
Emotional well-being	.59***	< .001	.37***	8.00	< .001
Self-esteem	.40***	< .001	–	–	–
Relationship to family	.31***	< .001	.03	.77	.44
Relationship to friends	.51***	< .001	.23***	6.00	< .001
Relationship to school	.39***	< .001	.05	1.18	.24
Total QoL	.58***	< .001	.08	.70	.49
**COVID school functioning**					
Experienced physical health	− .39***	< .001	.05	1.17	.24
Emotional well-being	− .42***	< .001	.02	.50	.62
Self-esteem	− .41***	< .001	–	–	–
Relationship to family	− .39***	< .001	− .07*	− 2.13	< .05
Relationship to friends	− .29***	< .001	− .13**	3.10	< .01
Relationship to school	− .52***	< .001	− .22***	− 5.12	< .001
Total QoL	− .54***	< .001	− .48***	− 4.27	< .001

*p < .05, **p < .01, ***p < .001.

**Table 3:** Correlations between the COVID-19 variables.

	Anxiety	Loneliness	School functioning
Anxiety	1	− .35***	.09**
Loneliness	− .35***	1	− .34***
School functioning	.09**	− .34***	1

*p < .05, **p < .01, ***p < .001.

## Discussion

This study aimed to investigate the impact of COVID-19 on the prevalence of traditional and digital bullying; emotional, behavioral, and peer problems; and QoL among children living in Northern Norway 1 year into the pandemic. Earlier studies, conducted during the first 3 months of the pandemic, are consistent in showing increased mental health problems and reduced QoL^4–6,8,16^. The studies that have explored bullying during the pandemic report more inconsistent results^35,36^.

### Prevalence of traditional and digital bullying

The prevalence of bullying during the pandemic was found to be 10.1% for traditional bullying and 6.0% for digital bullying. There were a significantly increased prevalence in both traditional and digital bullying during the pandemic compared to before the pandemic, which was only partly what was hypothesized. The prevalence from this study is higher than the recent national report from Bakken^27^, who reported 6% and 3%, respectively. This may be because this study measured prevalence both during the school day and outside of school combined. Bakken^27^ further reported that the prevalence of bullying was one percentage point lower than the year before. This corresponds with the findings of Vaillancourt et al.^35^, and it suggests that the prevalence of bullying reduced during the pandemic on a national level, which is also documented internationally. The current study, on the other hand, shows the opposite prevalence pattern in the city of Tromsø. This indicates that local differences are relevant on the impact of the pandemic on bullying and that consistent results may be difficult to obtain.

The increased prevalence of digital bullying that was found in this study is consistent with other findings from the pandemic^33,34^, and the results are concerning due to the known negative impact^31^. The overall findings on the prevalence of both traditional and digital bullying highlight the importance of implementing and maintaining anti-bullying measures on both national and local levels after the pandemic. Local requirements between areas and schools should be considered and emphasized.

In the pre-pandemic group, there were more males who reported being bullied (both traditional and digital) compared to females. During the pandemic there were no gender differences. The highest prevalence in both traditional and digital bullying was in grades 4 + 5. This is the period when most of the children reported being bullied. Further, the prevalence decreased through grades 6 + 7 and grades 8–10. The same prevalence pattern for the age groups was found in both the pandemic group and the pre- pandemic group. These patterns are also consistent with what other studies have documented^45^.

During the WiT project period (2012–2018), the participating schools placed high priority on anti-bullying measures and activities to increase well-being and life quality for the children at school. The activities were employed locally, by the school leaders, and were different for each school. Some of the schools had anti-bullying discussions and projects within each class. Some schools were mapping areas around the schools were bullying often took place and the teachers were asked to pay extra attention in these areas during their outdoor inspection time. Some of the schools arranged anti-bullying seminars for the teachers, where the parents also were involved. Results from analysis of the entire WiT project period suggested that the prevalence of both traditional and digital bullying decreased year by year. In the first year of the study 10.4% reported being bullied either traditional or digital^46^. In 2017 the prevalence had dropped to 8.1%. Thus, the prevalence of either traditional or digital bullying was higher during the pandemic, at 11.9%, compared to the entire WiT project period.

Previous studies have shown that increased presence and supervision from teachers and other school personnel reduced the prevalence of bullying during the school day^24^. During the pandemic, were school personnel assigned several more areas of responsibility than normal to maintain the infection control measures, such as hygiene measures, ensuring social distance between cohorts, follow-up of vulnerable and chronically ill pupils, and adapting and carrying out pedagogical measures and curriculum in a homeschool format^47^. Due to the isolation and quarantine regulations, the abrasion on the teachers have been sufficiently increased resulting in more absence and sick leave^48^. The shortage of teachers, also called the teacher crisis, left few resources or capacity to ensure a protective social environment for children and adolescents at school. As a result, the normal preventive measures against bullying may not have been maintained, giving the pupils more opportunities to bully.

### Emotional, behavioral, and peer problems

In line with earlier studies, increased mental health problems were found in the present study as well. The problems included increased emotional symptoms, conduct problems, and peer problems. Hyperactivity has also been reported as an increased behavioral problem during the pandemic ^7^, but in this study there was found no difference in the hyperactivity scale between the groups. In general, the females reported more difficulties than males, which also corresponds with earlier findings during the pandemic^4–6,8^. Females are more likely to develop a linear decrease in negative mental health problems, such as generalized anxiety disorder, panic disorder, and social anxiety disorder, whereas males are more stable^49^. Age group was also found to be a significant covariate in this study. Emotional symptoms increased with age, which is also in line with what was expected^50^. Peer problems were found to be highest in grades 4 + 5, which was also the age group where most children reported being bullied.

Already before the pandemic outbreak did a range of studies document an increase of emotional problems, clinical diagnostics, and treatment of children and adolescent psychiatric disorders in high-income countries over the past decade. Evidence from low- and middle-income countries are limited^51^. In Norway has the prevalence of self-reported mental health difficulties and registered psychiatric diagnosis increased among children and adolescence since 2010. A report from 2018 described increased emotional and behavioral difficulties each year, especially among females. The most common difficulties are anxiety, depressive, and eating disorder symptoms, but also increased academic performance pressure. In 2010, 7% of children and adolescence in Norway were registered with a psychiatric diagnosis in the primary health care sector in Norway. In 2020 this was increased to 10% and all difficulties tend to increase with age^52^. For example, Bakken^50^ report that at age 13 did 5% of males and 13% of females report that they were often bothered by depressive symptoms. At age 18, this was increased to 13% for males and 32% for females.

There are several known risk factors, besides gender, for developing emotional, behavioral and peer problems. Children and adolescents from families with low socioeconomic status, that have a difficult relationship with their parents, have adverse childhood experiences, or have few friends or poor peer relationships, report low well-being at school and/or are victims of bullying have increased risk of reporting mental health difficulties (51). The same risk factors have been associated with increased difficulties during the pandemic^4–6,8,16,27^ and highlight who are in most need of psychosocial support after the pandemic.

### The impact of anxiety, loneliness, and self-perceived school functioning on subjective life quality

Since mental health difficulties among children and adolescents were increasing already before the pandemic, it is important to highlight specific variables that has predicted reduced QoL during the pandemic. Increased anxiety, loneliness, and reduced QoL has been found in several studies from the pandemic^4–6,8,16^. In the present study we found that both anxiety (specifically about the spread of the virus) and loneliness due to reduced ability to socialize with friend, predicted reduced subjective QoL. Loneliness predicted reduced life quality in emotional well-being and relationship to friends, whereas anxiety predicted reduction in all the KINLD subscales.

Self-perceived school functioning was also found to be a strong predictor of reduced life quality in relationship to friends, family, school, and total QoL. To the best of our knowledge, this is not documented in other studies. Further, a medium correlation between loneliness and school functioning was also documented, which highlights the importance of support and perceived social well-being to function in school. Wang and Degol^22^ discussed the quality of interpersonal relationships, such as consistency, frequency, and nature of the relationships formed at school: pupil–teacher relationship, peer relationships, the relationship with the school staff, and the school–home relationship. Positive interpersonal relationships are important for school well-being, academic functioning, and performance. Connectedness, in terms of feeling acceptance, inclusion, belonging, and safety (both physical and emotional) in the school environment, is another important factor that may have been compromised during the pandemic.

### Limitations of the study

There are some limitations of the present study that need to be taken into consideration. First, the pre-pandemic group included data from 2017, when ideally the data should have been collected just before the outbreak of the pandemic. As discussed above, an increase of emotional problems was already documented before the pandemic. There may be other factors, in addition to the pandemic, that have influenced the prevalence of bullying and increased emotional, behavioral, and peer problems among the children and adolescents in this study that we have not accounted for.

There are also several biases associated with self-report data and since this study is purely based on self-report measures, we must acknowledge these limitations. First, there is a possibility that participants may have made answers that is more socially accepted among their peers rather than being truthful. All though this bias was addressed in the video that the participants watch before the data collection, there is a possibility that the data can have some degree of inaccuracy anyway. Second, the age range of the participants can be of concern because younger children can have problems in assessing themselves accurately or can have difficulties understanding the questions or how to respond to a self-report measure. This bias is addressed by using validated tools for the targeted age range, and during the data collection there was a teacher present that could help clarify possible confusions in the measure. There are several studies that indicate that children from 5 years of age can provide reliable and sound self-report^53–55^, however we do acknowledge that this is a possible limitation of the data.

Another limitation is that there are also a few differences in the data collection procedures between the pandemic group and the pre- pandemic group. The data from 2021 were collected anonymously, while the data from 2017 were collected from children providing their personal information; therefore, the data required written consent from one of the parents, and data lacking this consent had to be deleted from the material. There are studies that highlight the importance of anonymity to ensure reliable and honest reports on sensitive information such as mental health indicators, peer problems and victimization^56^. This indicate that the participants in the pre- pandemic group may to some degree have under-reported the severity of their mental health issues and peer problems.

The change in response rates between the groups (85% for the pandemic data versus 65% for the pre- pandemic data) may also introduce a bias into the measurements. Furthermore, the results may not be generalizable to all Norwegian children or adolescents, as the study was targeted to a single district in Northern Norway (Tromsø) and lacks the diversity of children and adolescents from across the country. However, the inconsistency in the literature on the prevalence of bullying suggests that results are difficult to generalize.

## Conclusion

This study documented increased prevalence of traditional and digital bullying and increased emotional, behavioral, and per problems among school children in Northern Norway during the COVID-19 pandemic compared to before. Most of the findings were in line with what was hypothesized, except for the increased prevalence in traditional bullying, which we hypothesized would be reduced. Further, anxiety, loneliness, and self-perceived school functioning were found to predict reduced subjective QoL. To the best of our knowledge, the ability to concentrate at school (both at school and during home-schooling) has not been documented as a predictor for reduced life quality in any other studies that have documented the impact of COVID-19 on children and adolescents.

### Implications of this study

The present study suggests systematically providing psychosocial and anti-bullying measures in schools after the pandemic. In Norway, children had a saying during the pandemic: “It will get well” (org. Alt blir bra). However, the findings from this study suggest that it will not get well by itself. In Norway, school owners are required by law to organize a healthy environment that secures and protects all children and adolescents with a safe physical and psychosocial environment^57^. Thus, we advise that both local and national government administrations and school owners implement systematic evidence-based psychosocial measures, increase the focus on the school climate and strengthen the teacher staff with more resources, such as a dedicated psychological adviser that can serve both pupils and teachers. Further, it would be beneficial to enhance and strengthen education on the negative impact of bullying, the impact of mental health issues on school functioning, and the prevention of mental health problems among children and adolescents. Teachers should aim to emotionally support their pupils, as well as provide educational support. Emotional support is especially important for pupils at risk of being vulnerable. Further research is necessary to investigate the implementation and effect of psychosocial and anti-bullying measures in schools. It would also be beneficial to include the experiences of children, parents, teachers, and school leaders to provide a comprehensive illustration of the need for and implementation of psychosocial measures.

The severe findings of increased bullying victimization in this study are an important contribution to a literature that report inconsistent findings^33–36^. Further, this study support earlier findings of increased emotional difficulties and reduced QoL during the lock-down period^4–6,8^, and document that this is also the case a year into the pandemic. Self-perceived school functioning has also been identified as an important predictor for QoL, which is a new finding to the existing literature. The methodology and approach of this study has added new perspectives and information on how children and adolescents have been affected by the pandemic.

## Supplementary Information

Supplementary Information.

## Data Availability

The datasets used during the current study are available from the corresponding author on reasonable request.
