# Supplementary material for: The severe impact of the COVID-19 pandemic on bullying victimization, mental health indicators and quality of life
Source: Sci Rep. 2022 Dec 31;12:22634. doi: 10.1038/s41598-022-27274-9 (PMC9804241; doi:10.1038/s41598-022-27274-9)
Supplement: Supplementary file 1 — Supplementary Information. [file 41598_2022_27274_MOESM1_ESM.docx]

**Appendix 1**

**Bullying:**

| **Verbal (α = 0.84)**  1. Have you experienced hurtful name calling?  2. Have you experienced hurtful name calling of someone in your family?  3. Have you experienced that someone has been mean and said that you are different?  4. Have you experienced being teased?  5. Has someone tried to hurt your feelings on purpose? | **Social (α = 0.85)**  1. Has someone gotten other children to be mean to you?  2. Has someone tried to get you to be mean to others?  3. Has someone tried to trick you to do something wrong?  4. Has someone tried to get you to do something that you didn’t want to do?  5. Has someone threatened to tell on you?  6. Has someone told a lie about you? |
| --- | --- |
| **Digital (α = 0.87)**  1. Has someone sent you creepy or unpleasant messages, pictures, or videos on the phone?  2. Have you experienced creepy or unpleasant calls on the phone?  3. Has someone sent you frightening e-mails?  4. Have you experienced being teased or insulted on social media?  5. Have you experienced being teased or insulted when playing digital games?  6. Have you experienced someone sharing unpleasant pictures or videos of you on social media?  7. Have you been shut out of closed groups on social media that you wanted to be included in? | **Physical (α = 0.86)**  1. Has someone tried to kick you?  2. Have you experienced being threatened?  3. Has someone tried to trip you?  4. Has someone tried to hit you? |

**SDQ:**

| **Emotional problems (α = 0.66)**  1. I get a lot of headaches.  2. I worry a lot.  3. I am often unhappy.  4. I am nervous in new situations.  5. I have many fears. | **Conduct problems (α = 0.60)**  1. I get very angry.  2. I usually do as I am told.  3. I fight a lot.  4. I am often accused of lying or cheating.  5. I take things that are not mine. |
| --- | --- |
| **Hyperactivity (α = 0.67)**  1. I am restless.  2. I am constantly fidgeting.  3. I am easily distracted.  4. I think before I do things.  5. I finish the work that I am doing. | **Peer problems (α = 0.41)**  1. I am usually on my own.  2. I have one good friend or more.  3. Other people my age generally like me.  4. Other children or young people pick on me.  5. I get on better with adults than with people my age. |
|  |  |

**KINDL:**

| **Experienced physical health (α = 0.63)**  1. I felt ill.  2. I had a headache or tummy ache.  3. I was tired and worn out.  4. I felt strong and full of energy. | **Emotional well-being (α = 0.68)**  1. I had fun and laughed a lot.  2. I was bored.  3. I felt alone.  4. I was scared. |
| --- | --- |
| **Self-esteem (α = 0.75)**  1. I was proud of myself.  2. I felt on the top of the world.  3. I felt pleased with myself.  4. I had a lot of good ideas. | **Relationship to family (α = 0.76)**  1. I got on well with my parents.  2. I felt fine at home.  3. We quarreled at home.  4. My parents stopped me from doing certain things. |
| **Relationship to friends (α = 0.74)**  1. I played with my friends.  2. Other kids liked me.  3. I got along well with my friends.  4. I felt different from other children. | **Relationship to school (α = 0.64)**  1. Doing my schoolwork was easy.  2. I enjoyed my lessons.  3. I worried about my future.  4. I worried about bad marks or grades. |

**COVID categories:**

| **Anxiety (α = 0.76):**  1. Have you been worrying about the virus?  2. Do you think about the virus even when you don’t mean to?  3. Have you been afraid to be infected when you are at school? | **School functioning (α = 0.73):**  1. Have you been able to concentrate on your schoolwork?  2. Did you manage to concentrate when there was homeschooling?  3. Have you been able to do your best with your schoolwork? |
| --- | --- |
| **Loneliness (α = 0.73):**  1. Have you been with your friends as much as you have wanted to?  2. Have you been lonely? |  |
